# Retinol kinetics in unsupplemented and vitamin A-retinoic acid supplemented neonatal rats: a preliminary model

**DOI:** 10.1194/jlr.M045229

**Published:** 2014-06

**Authors:** Libo Tan, Amanda E. Wray, Michael H. Green, A. Catharine Ross

**Affiliations:** * Graduate Program in Nutrition,Pennsylvania State University, University Park, PA 16802; † Department of Nutritional Sciences, Pennsylvania State University, University Park, PA 16802; § Huck Institutes for the Life Sciences, Pennsylvania State University, University Park, PA 16802

**Keywords:** neonate, liver, lung, compartmental model, WinSAAM, recycling

## Abstract

Vitamin A (VA) metabolism in neonates is virtually uncharacterized. Our objective was to develop a compartmental model of VA metabolism in unsupplemented and VA-supplemented neonatal rats. On postnatal day 4, pups (n = 3/time) received 11,12-[^3^H]retinol orally, in either oil (control) or VA combined with retinoic acid (VARA) [VA (∼6 mg/kg body weight) + 10% retinoic acid]. Plasma and tissues were collected at 14 time points up to 14 days after dose administration. VARA supplementation rapidly, but transiently, increased total retinol mass in plasma, liver, and lung. It decreased the peak fraction of the dose in plasma. A multi-compartmental model developed to fit plasma [^3^H]retinol data predicted more extensive recycling of retinol between plasma and tissues in neonates compared with that reported in adults (144 vs. 12–13 times). In VARA pups, the recycling number for retinol between plasma and tissues (100 times) and the time that retinol spent in plasma were both lower compared with controls; VARA also stimulated the uptake of plasma VA into extravascular tissues. A VARA perturbation model indicated that the effect of VARA in stimulating VA uptake into tissues in neonates is both dramatic and transient.

Very little is currently known about vitamin A (VA) metabolism in neonates in spite of the critical roles that VA plays in neonatal development. Specifically, VA is required for normal embryonic development, hematopoiesis, immune response, metabolism, and growth and differentiation of many types of cells (1, 2). VA is also necessary for both innate and adaptive immunity (3), and VA-deficient infants are at increased risk of mortality and infectious diseases (3–5). In addition, VA is important for normal postnatal development of the lung (6, 7). Studies in neonatal rat lung have indicated a significant accumulation and utilization of retinyl esters during the alveolar stage (8, 9) and an increase in retinol and retinoic acid (RA) in lung fibroblasts (10). The levels of retinoid binding proteins, RA receptors, and RA synthesizing enzymes have been shown to peak postnatally (11), suggesting a requirement for RA during alveologenesis.

In spite of the involvement of VA in so many physiological and metabolic systems, neonates begin life with low levels of VA (12). Comparison of VA levels in plasma, liver, and extrahepatic tissues in newborns with those in adults have consistently shown lower amounts in neonates, even in industrialized countries not known for VA deficiency. Plasma retinol levels in healthy newborns were ∼50% of the levels in the corresponding maternal plasma (13, 14), and concentrations of retinol-binding protein (RBP) and its cotransport protein transthyretin were also lower in newborns (7, 15). A liver retinol concentration of <10 μg/g tissue was reported in an autopsy study of otherwise healthy infants who died at 0–1 month of age from sudden infant death syndrome, as well as from other causes (16). Such a level is considered VA deficient in older children and adults. Similar values were reported in neonatal rats (17). VA stores are high in fetal lung and decrease toward term, and they are almost depleted in newborns (18, 19). Retinol levels are even lower in neonates in developing countries where VA intakes may be low and VA deficiency is a common and significant nutritional problem (20). Lower VA stores and plasma retinol concentrations are present in low birth-weight (LBW) infants and in preterm newborns, and poor VA status may contribute to their greater likelihood of developing chronic lung disease, such as bronchopulmonary dysplasia (21), a major cause of morbidity in preterm infants.

Currently, VA supplementation is included in the WHO/UNICEF Millennium Development Goals (22) as a strategy to reduce mortality in children aged 6–59 months in parts of the world where VA deficiency is a public health problem (23), and high-dose bolus VA supplementation has been widely adopted for this age group (24). However, studies investigating VA supplementation in 1–5-month-old infants did not show any survival benefits. Also, VA supplementation to newborns (0–28 days of life) has produced mixed results, with reduced mortality in some trials but not in others (25), although clinically, VA treatment has shown beneficial effects in elevating VA status, reducing bronchopulmonary dysplasia, and improving outcomes in LBW infants (26–28). As a result, the WHO has not yet established a position on VA supplementation in neonates (29). New trials are being initiated by WHO in Africa and Asia to determine if neonatal VA supplementation (50,000 IU given within the first days after birth) is effective in reducing morbidity and mortality (25).

Previously, we reported that oral administration of VA combined with RA (VARA), an admixture of VA and a small proportion (one-tenth) of RA, synergistically stimulates a rapid increase in retinol uptake and esterification in the lung of neonatal rats as compared with either VA alone or RA alone (30, 31). In addition, lung retinyl ester content was 5-fold higher in neonatal rats treated with VARA compared with VA alone, even in dexamethasone-treated neonatal rats (32). VARA was found to also partially ameliorate the effect of hyperoxia and attenuate oxygen-induced inflammation in neonatal lungs (33).

The use of methods of tracer kinetics and the application of compartmental analysis have led to an enhanced understanding of VA dynamics and metabolism in adult animals and humans, including prediction of extensive recycling of retinol among plasma, liver, and extrahepatic tissues, and estimates of the transit, turnover, storage, and utilization of retinol (34–39). However, compartmental ana­lysis has not yet been used to model VA metabolism in neonates. This may be in part because of the added challenges of applying this method to organisms that are not in a metabolic steady state. In the present study, we aimed to fill the gap by developing a compartmental model of VA kinetics in neonatal rats under different nutritional conditions (placebo and VARA). Information on the transfer, turnover, storage, and disposal of retinol in neonates was expected from the model as well as insight into the effect of VARA on VA kinetic behavior in a rat model of the neonatal life stage.

## METHODS AND MATERIALS

### Animals and diet

Animal protocols were approved by the Institutional Animal Care and Use Committee of the Pennsylvania State University. Sprague-Dawley female and male rats were housed with continuous access to food and water and a 12 h light/dark cycle. After mating, and throughout the study period, females were fed a diet with a marginal level of VA (0.35 mg retinol equivalents/kg diet; Research Diets, New Brunswick, NJ) to reduce the transplacental transfer of VA and the concentration of VA present in the dams’ colostrum and milk during lactation (17). Rat pups were assigned randomly to two treatments, a control (oil) group treated with canola oil to provide data on the kinetics of retinol in the absence of supplementation, and a VARA group to obtain the effect of acute VA supplementation.

### Preparation of the oral dose

Retinoids for the oral dose (all-*trans* retinyl palmitate and all-*trans* RA) were purchased from Sigma-Aldrich (St. Louis, MO). The amount of VA in the oral dose [∼6 mg/kg body weight (30)] was based on the amount of VA administered to human infants to reduce morbidity and mortality (50,000 IU/∼2.5 kg) (25). The amount of RA was determined in previous studies to stimulate retinol uptake and esterification in neonatal lungs (31) and to induce lung septation in neonatal rats (40). VARA was a mixture of the VA dose and the RA dose such that RA equaled 10% of the amount of VA. Canola oil was used as placebo. The VARA mixture, or oil only, was admixed with 11,12-[^3^H]retinol (Perkin-Elmer, Waltham, MA) so that the radioactivity in the oral dose was 0.2 μCi/μl. A volume of 0.8 μl/g body weight plus 1 μl for potential loss of the dose was given to each pup.

### Kinetic study

The dose was administered on postnatal day 4 (P4), which coincides with the time when alveolar septation begins in rats (41). The study continued throughout the period of maximum septation (until P14). On P4, each pup received a single oral dose containing 11,12-[^3^H]retinol, as above. The dose was delivered directly into the mouth via a small micropipette. After dosing, each tip was retained for extraction with hexanes. For pups that had any visible oil left on their muzzle, the muzzle was blotted with a small “chip” of paper towel which was extracted with the pipette tip. An aliquot of hexanes representing undelivered dose for each pup was taken for counting. In this way, the dose delivered to each pup was individually corrected for any losses. Aliquots of each dose preparation were extracted and analyzed for [^3^H]retinol to determine the value for 100% of the dose.

Immediately after dosing, the time was recorded and pups were returned to their mothers. Pups consumed mother’s milk throughout the study. Groups of pups (three/time/group) were euthanized at 14 time points after dosing, including several during the initial phase of VA absorption: 1, 2.5, 4, 6, 8, 11, 15, and 24 h, and 2, 4, 6, 8, 11, and 14 days. At these times, pups were weighed and euthanized with isoflurane (Phoenix Pharmaceutical, Saint Joseph, MO). Blood was collected from the vena cava into heparinized syringes, and tissues (liver, lungs, etc.) were excised and rapidly frozen in liquid nitrogen. Plasma was obtained following centrifugation of blood samples and stored at −20°C; tissue samples were stored at −80°C until analysis.

### Plasma analysis

An aliquot of each plasma sample (10–60 μl) was transferred into a vial containing 4 ml Scintiverse (Fisher Chemical) and analyzed for tritium content by liquid scintillation spectrometry; counting was done to a 1% counting error (38). Another aliquot of plasma (25–100 μl) was analyzed for total retinol concentration by ultra performance liquid chromatography (UPLC) as previously reported (30, 42).

### Tissue analysis

The extraction method for tissues was originally developed by Ross and Zilversmit (43) for lipid extraction and was based on the procedure of Thompson et al. (44). Briefly, portions of tissue were cut, weighed, and homogenized in 100% ethanol. Tissues were incubated in ethanol for at least 1 h and then lipids were extracted with 6 ml hexanes containing 0.1% butylated hydroxytoluene. After centrifuging, the upper phase was removed into new vials. Solvent was evaporated under argon. The extraction with hexane/butylated hydroxytoluene was repeated and the two extracts were pooled, after which the solvent was removed. A known amount of an internal standard, trimethylmethoxyphenyl-retinol, was added to 1 ml of extract; the samples were dried under argon and reconstituted in 200 μl of methanol for UPLC analysis of total retinol.

### Calculation of the fraction of the oral dose

The fraction of the ingested dose remaining in plasma at each sampling time for each pup was calculated as total radioactivity (dpm) in plasma divided by dpm in the ingested dose as determined for each pup. Total dpm in plasma was calculated from the measured plasma tracer concentration at each time × the estimated plasma volume, where plasma volume was calculated as the pup’s body weight × 0.035 ml plasma/g body weight. Radioactivity in the ingested dose was calculated as the dose total dpm minus the dpm remaining in the tip and paper chip combined.

### Model development

Model-based compartmental analysis was applied to the plasma tracer response profiles (the mean fraction of the ingested dose at each time within each group versus time after dose administration) for neonatal rats in both groups using the Windows version of the Simulation, Analysis, and Modeling software (WinSAAM) (45). A proposed multi-compartmental model of VA kinetics (“proposed model” ; see Results) was developed based on a previous model proposed by Cifelli et al. (46), which describes the metabolism of orally administered VA in humans, including the absorption phase. Plasma tracer data were analyzed in light of the proposed model using WinSAAM until a satisfactory fit was obtained between observed and model-predicted values. Specifically, model parameters {fractional transfer coefficients [L(I,J)s]; see below} and, when necessary, model structure, were iteratively adjusted until a close fit was obtained, as judged by visual inspection of the simulated tracer data plot and by statistical analysis, including the sum of squares from nonlinear regression analysis and the estimated fractional SD (FSD) for each kinetic parameter. An F-statistic (47) and the Akaike information criterion (48) were used to statistically test whether increases in model complexity were justified. The model complexity (and thus the number of pa­rameters) was increased only when it resulted in a significant improvement in the sum of squares as determined by an F-statistic and reduced Akaike information criterion by more than 1–2 units. Once a satisfactory fit was achieved, the final estimates of the L(I,J)s and their statistical uncertainties were generated by nonlinear regression analysis in WinSAAM. For weighting purposes, an FSD of 0.05 was assigned to each datum. Parameters were considered well-identified if their estimated variability (FSD) was less than 0.5.

### Model-predicted kinetic parameters

L(I,J) is the fraction of retinol in compartment J transferred to compartment I each day. L(I,J)s are parameters that define the behavior of the system. Although neonatal rats were in a metabolic nonsteady state due to growth, we hypothesized that the fraction of retinol that was transferred between compartments was not changing with time. Thus, we set L(I,J)s to be time-invariant. The following parameters were calculated from model-generated L(I,J)s (49): mean transit time [t(I)] or turnover time is the mean of the distribution of times that a retinol molecule entering compartment I spends there during a single transit before leaving reversibly or irreversibly; mean residence time [T(I,J)] is the average of the distribution of times that a retinol molecule spends in compartment I before irreversibly leaving it after entering the system via compartment J; recycling number [*ν*(I)] is the average number of times a retinol molecule recycles through compartment I before it irreversibly exits from compartment I; fractional catabolic rate (FCR) is the fraction of the retinol pool which is utilized each day; disposal rate (DR) is the rate of irreversible utilization of retinol; traced mass [M(I)] is the amount of retinol in compartment I; and transfer rate [R(I,J)] is the amount of retinol transferred from compartment J to compartment I each day and is calculated as the product of M(I) and L(I,J). Parameters were then compared between the control group and the VARA group. A VARA perturbation model was also developed by applying a nonsteady state solution in WinSAAM to estimate the perturbation that VARA exerts on the system, as further presented in the Results.

### Statistical analysis

Data for tissue VA mass and tracer responses are reported as mean ± SEM. Differences among groups, *P* < 0.05, were determined by two-way ANOVA followed by a Bonferroni post hoc test using Prism software (GraphPad, La Jolla, CA). Compartmental modeling was done using group mean data at each time [“super-pup” model (35)]. For kinetic parameters, L(I,J)s are presented with estimated FSDs. Differences of L(I,J)s between groups, *P* < 0.05, were determined by using an unpaired *t*-test. Briefly, *t*-statistic for each L(I,J) was calculated using the equation (Value_1_ − Value_2_)/sqrt(SEM_1_^2^ + SEM_2_^2^), which was then applied to the *t*-table to determine *P* < 0.05, with df = 60 based on (3 pups/time × 14 times − 12 parameters) × 2 treatments.

## RESULTS

### Animal growth

**Fig. 1.**
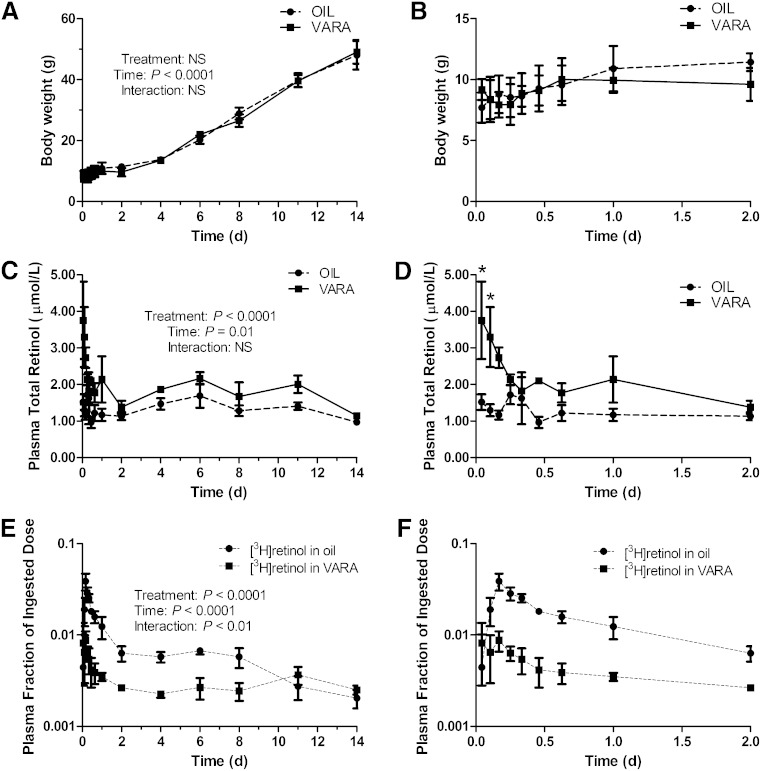
Body weights of pups (A, B), concentrations of total retinol in plasma (C, D), and fraction of administered dose in plasma versus time (days) after administration of [^3^H]retinol in oil or in VARA to neonatal rats. Plots of data from the first 2 days after dose administration, expanded from (A), (C), and (E), are shown in (B), (D), and (F). Points shown are means ± SEM, n = 3 per time per group. Results of two-way ANOVA for each factor (treatment, time, and interaction) are shown in (A), (C), and (E). *Significant differences between VARA and oil-treated pups at the same time point as indicated (*P* < 0.05).

The body weights of the pups versus time after dosing are shown in **Fig. 1A, B**. Body weight was relatively constant during the first 2 days after dosing, when pups were 4–6 days old. Pups started to grow robustly 2 days after dosing. VARA treatment did not affect pup body weights.

### VA mass in plasma

In the control group, plasma retinol concentration (Fig. 1C, D) was relatively constant (0.9–1.5 μM) throughout the study period. Plasma retinol was significantly increased (*P* < 0.01) in the VARA group at 1 and 2.5 h after dosing compared with the oil group, such that levels were 3- to 4-fold higher in VARA-treated rats (Fig. 1D). Plasma retinol in the VARA group then decreased gradually to a level similar to that in the control group by 2 days after dosing. After 2 days, plasma retinol concentrations were comparable in the two groups.

### Kinetics of plasma VA

Data on the fraction of the dose remaining in plasma versus the time after dose administration are plotted semi-logarithmically in Fig. 1E, F. In the control group, radioactivity in plasma rose quickly during the first 4 h after dose administration, with the absorption peak (3.9% of the administered dose) at 4 h. Thereafter, the tracer rapidly disappeared from plasma followed by a leveling off of the plasma decay curve, which reflects the recycling of retinol between plasma and extravascular tissues. Following a constant period from 2 to 8 days after dosing, radioactivity further declined from 8 to 14 days into a terminal slope, which represents the disposal (utilization) of retinol. For the VARA-treated pups, the fraction of the dose remaining in plasma was much lower, although the pattern of tracer response was similar to that in the control group, with the absorption peak also at 4 h, but at only 0.9% of the dose. Plasma radioactivity was nearly constant in the VARA group from 2 days to the end of the study.

### Model development and proposed model

**Fig. 2.**
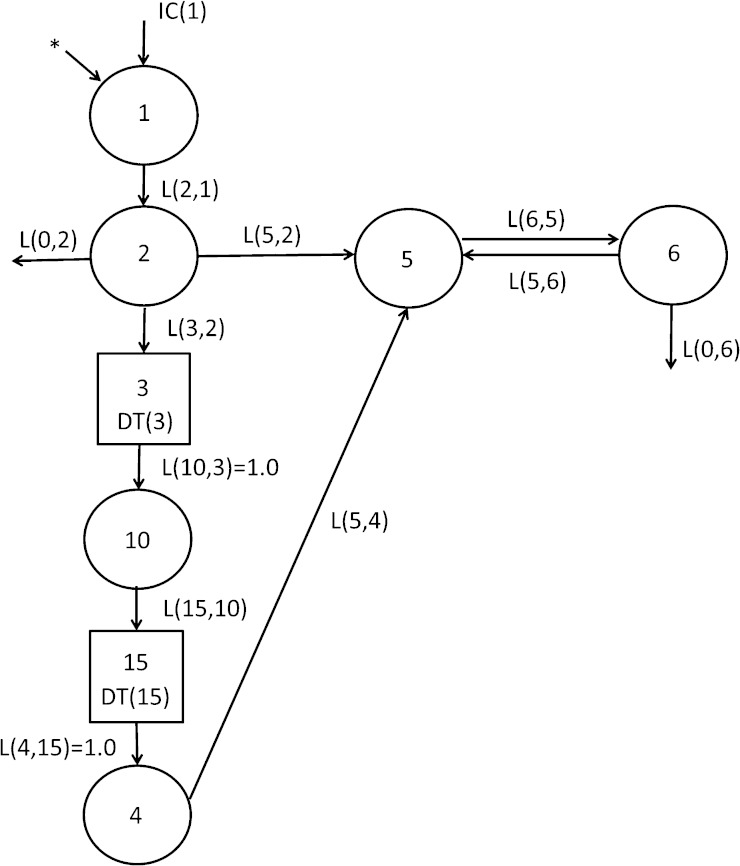
Proposed multi-compartmental model for VA metabolism in neonatal rats after administration of [^3^H]retinol in oil or in VARA based on plasma tracer response data versus time. Compartments are represented as circles; interconnectivities between compartments correspond to L(I,J)s, or the fraction of retinol in compartment J transferred to compartment I per day; components 3 and 15 are delay elements. Compartments/components 1–3 represent VA digestion and absorption. Compartments/components 10, 15, and 4 represent chylomicron metabolism, the uptake of chylomicron remnants, and the processing of VA in extravascular tissues. Compartment 5 represents plasma retinol bound to RBP; this retinol exchanges with VA in one extravascular pool (compartment 6). The asterisk represents the site of input of [^3^H]retinol and is also the site of input of dietary VA.

**Fig. 3.**
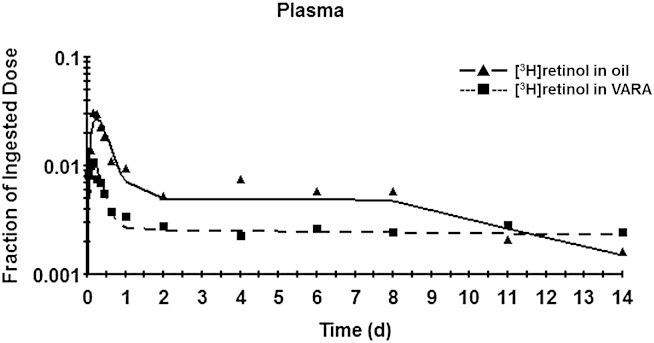
Mean observed (symbols) and model-predicted (lines) fraction of administered dose in plasma versus time (days) after administration of [^3^H]retinol in oil or in VARA to neonatal rats. Plot of data from the first 2 days after dose administration, expanded from (A), is shown in (B). Each point represents the mean of n = 3 pups.

A multi-compartmental model for VA kinetics in neonatal rats, shown in **Fig. 2**, was developed based on the plasma tracer response profiles (Fig. 1E). The flow from compartment 1 (the site of input of [^3^H]retinol and dietary VA) to compartment 4 represents the process of VA digestion, absorption, chylomicron production, and metabolism; compartments 1 and 2 can be viewed as the gastrointestinal tract. Delay element 3 corresponds to chylomicron production before chylomicrons are secreted into plasma, compartment 10 represents newly absorbed retinyl esters in plasma chylomicrons, and delay element 15 corresponds to chylomicron metabolism before the uptake of retinyl esters into liver and other tissues, which is represented by compartment 4. After the processing of VA, retinol-RBP is secreted into plasma (compartment 5). Compartment 5 represents the retinol-RBP pool in plasma; this VA exchanges with one extravascular VA pool (compartment 6); compartment 6 is the site of irreversible loss of VA. Because [^3^H]retinol was given orally, the radioactivity detected in the plasma presumably comes from both retinyl esters in chylomicrons and retinol-RBP. Accordingly, the plasma tracer response was assigned to compartment 10 plus compartment 5. L(5,2) reflects the possibility of direct secretion of retinol-RBP from the enterocytes into plasma compartment 5. L(0,2) represents the fraction of the tracer that is not absorbed. Based on visual inspection, it is evident that the proposed model provided a good fit to plasma tracer data for both the control group and the VARA group (**Fig. 3**).

### Model-derived kinetic parameters

L(I,J)s and their estimated FSDs for the models for both groups are listed in **Table 1**. L(I,J)s related to plasma retinol kinetics [L(5,4), L(6,5), L(5,6), and L(0,6)] were well-identified (FSD <20%), whereas L(I,J)s involved in VA absorption and chylomicron metabolism [L(2,1), L(0,2), L(5,2), L(3,2), and L(15,10)] were not identified with certainty, as indicated by their large FSDs. The uncertainty in these L(I,J) values indicates that these parameters could not be adequately identified using the present data collected in a study that did not focus on the process of VA absorption and chylomicron metabolism. L(0,2), the fraction of the dose that was not absorbed, was very small compared with the value for [L(5,2) + L(3,2)], the fraction absorbed, in both groups, indicating that the absorption efficiency of VA in neonatal rats might be very high. L(15,10), which characterizes the fraction of retinyl esters in chylomicrons/chylomicron remnants that is taken up/cleared from plasma by liver and other tissues, was higher in the VARA group than in the oil group. L(6,5), the fraction of retinol in compartment 5 (plasma retinol-RBP) that was transferred to compartment 6 (extravascular VA) per day was significantly higher in the VARA group than in the oil group. The higher values for L(15,10) and L(6,5) in VARA-treated neonates was consistent with the lower fraction of dose in plasma (shown in Fig. 1E). L(0,6), which corresponds to the irreversible loss of retinol, was low in both groups. This finding is consistent with visual examination of the plasma tracer response curves; in that there is a plateau period from 2 to 8 days in the control group and from 2 days to the end of the study in the VARA group. Because the plasma tracer response profile after 8 days was steeper in the oil group, indicating a change in retinol disposal/utilization after 8 days, we applied an option called “time interrupt” in WinSAAM to set a new value for L(0,6) after 8 days, as indicated in Table 1. The value for L(0,6) in the oil group was smaller (0.004) before 8 days and larger (0.197) after 8 days.

**TABLE 1.** Model-predicted L(I,J)s

	Value (FSD)	
L(I,J)	Oil (day^−1^)	VARA (day^−1^)
L(2,1)	5.09	11.5
L(0,2)	0.43	0.02
L(5,2)	0.44	0.00
L(3,2)	18.6	1980
L(15,10)	82.6	647
L(5,4)	5.06 (0.14)	5.52 (0.07)
L(6,5)	60.3 (0.07)	169.4 (0.04)*^a^*
L(5,6)	0.60 (0.10)	0.83 (0.05)*^a^*
L(0,6), before day 8	0.004 (0.79)	0.008 (0.45)
L(0,6), after day 8	0.20 (0.06)	0.008 (0.45)*^a^*

Shown are model-predicted L(I,J)s or fraction of retinol in compartment J that is transferred to compartment I each day (estimated FSDs in parentheses). The model is shown in Fig. 2.

a Significant differences (*P* < 0.05) from the placebo group.

Mean transit times, residence times, FCRs, and recycling numbers for retinol in both groups are shown in **Table 2**. The calculated transit time in plasma [t(5)] and residence time in plasma [T(5,5)] for the control group indicate that a retinol molecule spends 0.4 h in plasma during a single transit and 2.4 days in plasma before irreversible loss. VARA treatment decreased both the transit time and the residence time of retinol in plasma. The estimated ν(5), the number of times a retinol molecule recycles through plasma, indicated that retinol recycles 144 times through plasma before irreversible loss in control (oil-treated) neonatal rats. VARA supplementation decreased the recycling number to 100.

**TABLE 2.** Model-predicted mean transit times, residence times, FCRs, and recycling numbers in neonatal rats dosed with [^3^H]retinol in oil or VARA

Parameters	Oil	VARA
t(5), h	0.40	0.14
t(6), days	1.64	1.19
T(5,5), days	2.40	0.60
FCR(5,5), day^−1^	0.42	1.67
FCR(5,5), day^−1^ (after day 8)	14.7	1.67
ν(5)	144	100

Parameters shown are: mean transit time [t(5)], or the mean of the time that a retinol molecule spends in compartment 5 (plasma retinol-RBP) during a single transit before leaving reversibly or irre­versibly; mean transit time [t(6)], or the mean of the time that a retinol molecule spends in compartment 6 (extravascular tissue retinol) during a single transit before leaving reversibly or irreversibly; mean residence time [T(5,5)], or the average of the distribution of times that a molecule of retinol spends in compartment 5 (plasma retinol-RBP) before it leaves irreversibly; FCR(5,5), or the fraction of the plasma retinol pool which is utilized each day; recycling number [ν(5)], or the average number of times a retinol molecule recycles through com­partment 5 (plasma retinol-RBP) before it irreversibly exits from it. The model is shown in Fig. 2.

The model-predicted retinol DR, reflecting the utilization of retinol, is shown in **Table 3** for control pups. Because total body retinol mass increases with the growth of pups, the DR also varies with time, and thus, we calculated DRs at each time. The DR was low before 8 days and increased greatly to ∼20–30 nmol/day after 8 days, when the terminal slope of the plasma tracer response curve was steeper (Fig. 3). Note that retinol turnover rates in the oil group were much higher than their DRs.

**TABLE 3.** Calculated retinol DR and turnover rate [R(6,5)] for neonatal rats in the control group

Days After Dosing	DR (nmol/day)	Turnover Rate (nmol/day)
0.042	0.17	23.8
0.104	0.15	21.7
0.167	0.15	21.8
0.250	0.23	32.5
0.333	0.19	27.6
0.458	0.13	18.6
0.625	0.16	23.3
1	0.19	26.7
2	0.19	27.2
4	0.29	42.3
6	0.50	72.4
8	0.54	78.0
11	29.2	117
14	24.0	96.2

Shown are calculated retinol DRs and turnover rates for neonatal rats in the control group at 14 time points after dosing. The oral dose was delivered on P4. The DR is equal to R(0,6), the amount of retinol lost from compartment 6 (extravascular VA; Fig. 2) each day. The turnover rate is equal to R(6,5), the amount of retinol transferred from compartment 5 (plasma retinol-RBP) to compartment 6 (extravascular VA) each day. R(0,6) and R(6,5) were both calculated by WinSAAM by including an estimate of the plasma retinol pool size [M(5)] in the model. Plasma retinol pool size was obtained from the UPLC results and estimated plasma volume.

### The VARA perturbation model

**Fig. 4.**
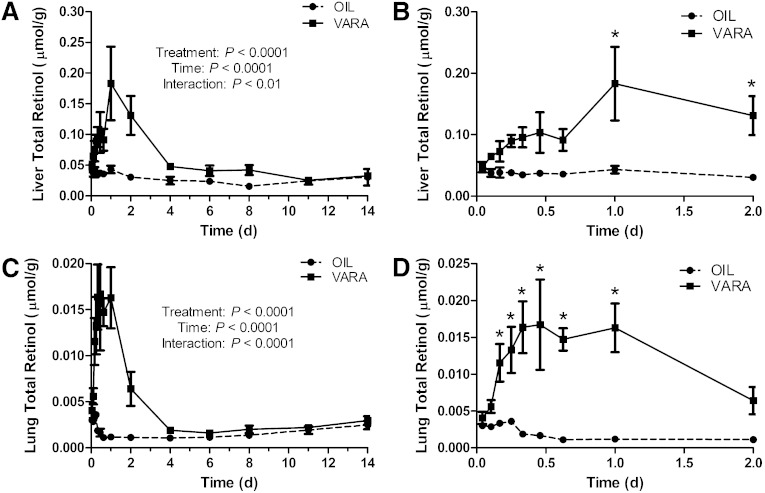
Concentrations of total retinol in liver (A, B) and lung (C, D) versus time (days) after administration of [^3^H]retinol in oil or in VARA to neonatal rats. Plots of data from the first 2 days after dose administration, expanded from (A) and (C), are shown in (B) and (D). Points shown are means ± SEM, n = 3 per time per group. Results of two-way ANOVA for each factor (treatment, time, and interaction) are shown in (A) and (C). *Significant differences between VARA and oil-treated pups at the same time point as indicated (*P* < 0.05).

The comparison of parameters for the compartmental model in the control group versus the VARA group indicated one very notable effect of the VARA treatment, an increase of approximately three times in the value of L(6,5), the fraction of plasma retinol transferred to extravascular tissues per day. As shown by analysis of tissue retinol concentration, the effects of the one-time VARA treatment were quite transient. Specifically, retinol masses in plasma (Fig. 1C, D), liver, and lung (**Fig. 4**) increased dramatically in the first 1–2 days after VARA administration; however, they weakened gradually and disappeared after 4 days. As a result of this observation, we hypothesized that the stimulating effect of VARA on L(6,5) might not last through the study period and thus might be time-variant. We therefore developed a “VARA perturbation model” to reflect the transient and time-dependent effect of VARA on the uptake of plasma VA into tissues. Whereas the compartmental model in Fig. 3 for the VARA group is still meaningful in providing information on the average effect of treatment on the system, the perturbation approach could improve understanding of the effects of VARA on retinol kinetics.

**Fig. 5.**
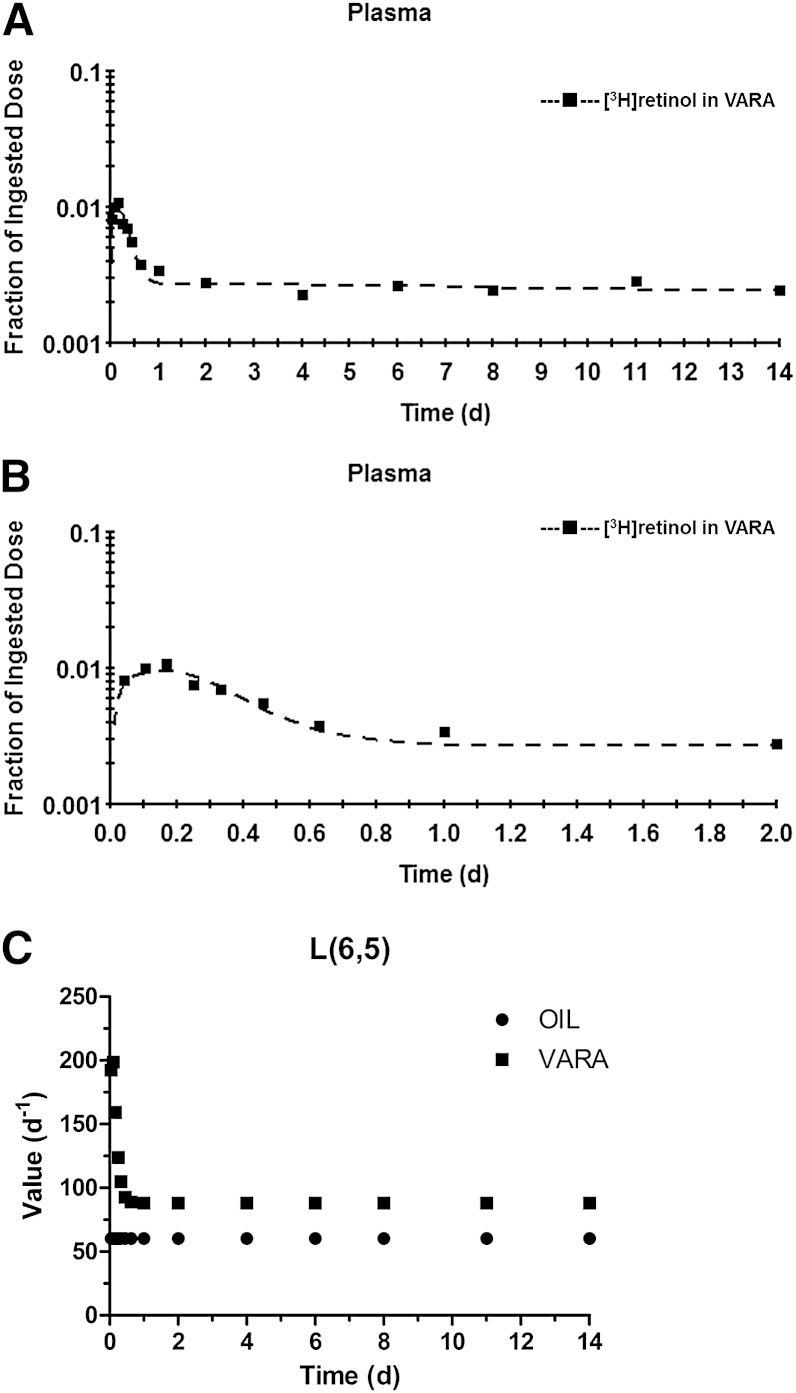
A, B: Mean observed (symbols) and VARA perturbation model-predicted (lines) fraction of administered dose in plasma versus time (days) after administration of [^3^H]retinol in VARA to neonatal rats. Plot of data from the first 2 days after dose administration, expanded from (A), is shown in (B). Each point represents the mean of n = 3 pups. C: Values for L(6,5) [the fraction of retinol in compartment 5 (plasma retinol-RBP) that is transferred to compartment 6 (extravascular tissue VA) per day] from the compartmental model for the control group and from the VARA perturbation model versus time (days) after administration of [^3^H]retinol in oil or in VARA to neonatal rats.

The structure of the perturbation model is same as that of the compartmental model in Fig. 3, except that an exponential equation was included for the VARA group to describe the effect of time on L(6,5). The equation, which was based on the shape of the tracer response curve, was L(6,5) = K × [K(11) × (e^−P(1)T^ − e^−P(2)T^) + K(12)], where T is the time after dose administration and K, K(11), P(1), P(2), and K(12), together with other L(I,J)s, are parameters for the model to fit the plasma tracer response in WinSAAM. As shown in **Fig. 5A, B**, the perturbation model provided a good fit to plasma tracer data for the VARA group. The sum of squares from weighted nonlinear regression analysis by WinSAAM is 1.85 × 10^−6^. The final values for K, K(11), P(1), P(2), and K(12) were 1.1, 450, 10.1, 19.6, and 80, respectively, and yielding the equation L(6,5) = 1.1 × [450 × (e^−10.1T^ − e^−19.6T^) + 80]. Values for L(6,5) versus time after dosing predicted by the perturbation model for the VARA group are listed in **Table 4** and plotted in Fig. 5C, in comparison to the value of 60.3 for L(6,5) in the compartmental model in the oil group (Table 1). The value of L(6,5) was increased in neonates treated with VARA from 60.3 to 190–200 during the first 2.5 h. The value of L(6,5) remained high in the first 4 h, then gradually decreased and reached a level similar to that for the control group at 1 day; it then stayed constant at 88 until the end of the study. That is, the effect of VARA on L(6,5) was dramatic but transient, which is consistent with its effect on retinol mass in plasma, liver, and lung.

**TABLE 4.** Values for L(6,5) from the VARA perturbation model

Time (days)	L(6,5) (day^−1^)
0.042	193
0.104	199
0.167	159
0.250	124
0.333	105
0.458	92.7
0.625	88.9
1	88.0
2	88.0
4	88.0
6	88.0
8	88.0
11	88.0
14	88.0

Shown are L(6,5) [the fraction of retinol in compartment 5 (plasma retinol-RBP) that is transferred to compartment 6 (extravascular tissue VA) per day] for the VARA perturbation model at 14 time points after dose delivery. L(6,5) for the VARA groups was calculated from the equation L(6,5) = 1.1 × [450 × (e^−10.1T^ − e^−19.6T^) + 80], which was obtained from the perturbation model. L(6,5) for the compartmental model for the control group is 60.3.

## DISCUSSION

In this study, a multi-compartmental model of VA metabolism and kinetics in neonatal rats was established for the first time by applying model-based compartmental analysis. The proposed eight compartment model (Fig. 2) provides a good fit for the plasma tracer response in both groups.

Our model predicts that digestion and absorption of VA might be very efficient in neonatal rats, as in adults (50), and very rapid. Digestion of VA depends on adequate pancreatic function and adequate lipid intake for micellization of the retinol. The exocrine pancreas is immature in newborns (51). However, because fat, which is present in large amounts in milk, is digested well by neonates, it seems likely that a combination of lipases from the pancreas, intestine, and breast milk are sufficient for the digestion of retinyl esters (52). Also, neonates utilize lingual lipase for fat digestion (53). Previous research has shown that levels of important proteins involved in intestinal retinyl ester formation, including cellular RBP type II, CRBP2, and lecithin:retinol acyltransferase (LRAT) increase at birth, reach high levels in the middle of the suckling period, and then decline after weaning (54). The capacity for absorption of VA, even at the supplemental level of 6 mg/kg that was used with VARA and which corresponds to the dose used in VA supplementation programs in young children, appears to be well-developed in the neonate. It is important to note that the present study was principally designed to describe the kinetics of plasma retinol (i.e., transit, turnover, recycling, and utilization of retinol-RBP) rather than VA digestion, absorption, and chylomicron metabolism. The absorption part of the model was necessary because we chose to orally administer the [^3^H]retinol dose, both due to the technical challenges of working with neonates and because the oral route of VA supplementation is physiological. The inclusion of the absorption part of the model, which is completed quickly, serves to deliver the tracer into compartment 5 (plasma retinol-RBP). To obtain FSDs for the L(I,J)s related to plasma retinol kinetics [L(5,4), L(6,5), L(5,6), and L(0,6)], the focus of the present study, we fixed some of the L(I,J)s in the absorption part of the model at the values that collectively gave the best fit to the early plasma data. Sensitivity analysis indicated that fixing these absorption L(I,J)s at slightly different values would not affect estimates of the retinol kinetic parameters. Changing fixed values of absorption L(I,J)s by 50% resulted in changes in L(6,5) of no more than 2% and in L(5,4), L(5,6), and L(0,6), values of which are relatively small, of no more than 5%. As mentioned earlier, further kinetic experiments focusing on the period of VA absorption in detail will need to be carried out before any conclusions can be drawn regarding the kinetics of the absorption process.

The model indicates that there is extensive recycling of retinol among liver, plasma, and extrahepatic tissues in neonates, as has been observed in adults (34), and that the plasma retinol turnover rate is much higher than the retinol DR (Table 3). The system of VA metabolism in neonates is therefore a high response system, as it is in adults (37). This means that, because the plasma retinol turnover rate is much higher than the DR, if tissue demands for retinol increase, recycling could decrease without an appreciable change in plasma retinol level (37). The recycling of retinol in neonatal rats is, however, much more extensive than that in adults and the retinol turnover is faster. The recycling number is ∼12–13 in adult rats (37), compared with 144 in these oil-treated neonatal rats (Table 2). L(6,5), the fraction of plasma retinol that is transferred to extravascular tissues per day, is ∼60 in oil-treated neonates. In similar models for adult rats, the value for this parameter is ∼10 (37). The time that retinol spends during a single transit in plasma is 1.9 h in adults (37), while it is 0.4 h in neonates. The faster turnover of retinol might be an adaptive mechanism to the lower VA level in neonates as mentioned. It might make retinol more available when tissue demands for retinol increase. The molecular mechanism involved in the faster retinol turnover in neonates is unknown.

The current compartmental model was developed based on plasma kinetic data, the patterns of which were well-defined in both groups. The curves had both an upswing representing mostly absorption, and a downswing representing mostly equilibration of plasma retinol with tissue retinol pools. The leveling off of the decrease in the fraction of dose between 1 day and 2 days indicates the recycling of [^3^H]retinol between plasma and tissues. The plateau after 2 days is an interesting finding which we cannot explain, except to note that the retinol utilization rate might be low during this period in preparation for later demands. A steep terminal slope, which indicates the disposal/utilization of retinol, was observed in the control group after 8 days. However, in the VARA group, the plateau period lasted until the end of the study. It is possible that the FCR, which represents the turnover of “endogenous” retinol (i.e., that which the oil group derived from the milk of the VA-marginal dams) was lower in the VARA-treated pups than that in the oil group. Another possibility is that a terminal slope would appear after 14 days and thus an extended experimental period might be considered for a future study so that VA DRs for VARA-treated pups could be calculated.

In the present study, the average effects of VARA were obtained from the comparison of parameters of the compartmental model between the two groups; a nonsteady state solution was applied in the VARA perturbation model to estimate the perturbation that VARA exerts on the system; the effects of the treatment were also obtained from the data for tissue VA masses and comparison of tracer response profiles.

In this study, neonatal rats were nursed by dams fed a marginal VA diet to resemble the VA status of at-risk newborn infants in parts of the developing world or in LBW infants in the US. Based on measurement of total retinol concentration in plasma and liver, the oil-treated pups had a marginal to adequate plasma VA level (Fig. 1C, D) according to the criteria for adults (55), while the liver retinol mass (Fig. 4A, B) indicated a marginal VA status, as expected. Measured plasma VA level in pups is similar to that in newborn human infants as previously reported (13). VARA supplementation had a dramatic and rapid effect on increasing the retinol mass in plasma, liver, and lung. Liver retinol concentration was significantly higher (*P* < 0.01) 1 day and 2 days after dosing in the VARA group (Fig. 4B). VARA increased lung total retinol in the first 4 days after dosing (*P* < 0.05 versus control at 4, 6, 8, 11, 15 h, and 1 day after dosing; Fig. 4D). However, the effect of the one-time VARA treatment was transient as, by 2 days after dosing, there was no evidence of supplementation on plasma retinol, while the effect of VARA supplementation on tissue retinol concentration disappeared after 4 days in the liver and lung. Previous reports on children 6–59 months of age also indicated that one-time high dose VA supplementation had a positive but transient effect on serum retinol level (56).

The VARA perturbation model also indicated that the perturbation that VARA exerted on the system was rapid and short-term; it was dramatic in the first 8 h after dose administration and then decreased gradually. The VARA treatment in our experiment was a one-time dose given with the [^3^H]retinol and therefore the transient effects were expected. We hypothesize that repeated doses of VARA would maintain these stimulatory effects; this has been proved in neonatal lungs in one recently published study of our laboratory (57), showing that lung retinyl esters in neonatal rats increased cumulatively with multiple dosing of VARA on P4, P7, P11, and P14.

It has been reported that VARA upregulates the mRNA expression of stimulated by RA gene 6 (STRA6), which is responsible for the uptake of retinol, and LRAT, which esterifies retinol, in neonatal lung at 6 h after retinoid administration (58). An increase in STRA6 and LRAT might explain why lung retinol mass was rapidly stimulated by VARA treatment. The stimulatory effects of VARA on these genes disappeared at 12 h after dose administration (58), which might be a reason why the effect of one-time VARA dosing on increasing retinol mass in neonatal lung ceased to be evident after 1–2 days.

The model also predicted that VARA decreased the transit time and residence time of retinol in plasma, and decreased the recycling number for retinol between plasma and tissues in neonatal rats. The last two findings are consistent with the prediction that VARA directed more retinol into tissues and might stimulate the storage of VA in these tissues, so that each retinol molecule spent less time in plasma and retinol recycling was decreased.

The present work may contribute to research in the fields of public health nutrition and clinical nutrition to solve VA-related problems in neonates. The current Dietary Reference Intake (DRI) for VA in infants was calculated from the VA content of human breast milk, rather than from knowledge of the true VA requirement in infants, which is still unknown. The calculation of the VA DR in this study, which is 20–30 nmol/day after 8 days in oil-treated neonatal rats, is related to the measure of actual requirements, so the nutritional requirement for VA in neonates can be better estimated. The current DRI for VA in infants of 0–6 months is 400 μg/day (1.40 μmol/day). Zachman (59) calculated retinol intake by a healthy 3.5 kg term neonate based on the retinol content in breast milk and a minimum daily intake of 1.47–2.93 μmol retinol/day was indicated. Rat pups at 11 and 14 days after dosing (ages P15 and P19) in our experiment weighed ∼45 g. Converting the result of the calculated DR (20–30 nmol/day) in a 45 g pup to that in a 3.5 kg infant, the DR is 1.56–2.33 μmol retinol/day. This is in the range of the retinol level that breast milk supplies according to the calculation by Zachman (59), but it is higher than the current DRI.

The compartmental model established in this study can be used as the initial model for developing compartmental models of VA dynamics in neonatal rats under different conditions, from which the effects of these conditions can be explored. Interesting conditions for further study include differences in VA statuses, various VA or non-VA supplementation strategies, perturbation by exogenous factors, and diseases that affect VA metabolism. The present model that was developed based on only plasma data can also be of translational value in developing compartmental models of VA kinetics in infants and children, because no organ data are available in human kinetic studies. In addition, because the dosage of VA was comparable to that given to infants in public health studies, the findings regarding the effects of VARA may be helpful in establishing guidelines for field- or hospital-based VA intervention programs.

There are several limitations to the present model. One is the uncertainty of the absorption part as mentioned earlier. A second potential limitation is the use of the “time-interrupt” option for the plasma kinetic data after day 8 in the control group; although we note that the time-interrupt feature has been used in several previous modeling studies, when it would improve the model (36, 60–63). At present, we feel confident that its use improves our model, but we do not have a biological explanation for why it is needed. Possibilities include a change in the rate of retinol oxidation beginning around day 8 (P12), which can be tested in future studies. A third limitation is that we did not include the tracee model for specific tissues here, as it will be the focus of a study on the contribution of individual tissues to whole-body retinol kinetics in the future. A potential strength of the present work is that it demonstrates an approach based only on analysis of plasma retinol kinetics that could be translated in the future to human studies.
